# News and Notes

**Published:** 1898-11

**Authors:** 


### *NEWS AND NOTES.*

---

Dr. J. W. Duncan, of this city, who, after a very severe illness, went to the mountains of Tennessee to recuperate his lost strength, is again at the post of duty.

---

Dr. James N. Ellis, formerly of Richmond, Va., but who for the last two years has been studying abroad, has located in Atlanta to engage in the practice of his profession.

---

Dr. G. G. Roy was elected as councilman from the Sixth ward at the city primary on October 5th. This the same as an election, and Dr. Roy will represent his ward for the next two years.

---

Dr. L. A. Felder, Secretary of the Atlanta Society of Medicine, has accepted and entered upon the duties of Contract Surgeon, U. S. V. Dr. S. A. Visanska has been appointed Secretary *pro tem.*

---

Dr. S. G. C. Pinckney spent the month of September in New York, his old home, enjoying a well-earned vacation. He also spent some time in the hospitals, seeing what was new in nervous diseases.

---

Dr. Park Howell, of this city, who has been acting as Assistant Surgeon in the Volunteer Army, has been detailed for duty at Santiago, Cuba. He left the city on October 1st for his new field of labor.

---

Dr. M. A. Purse was married on September 27th to Miss Josephine Earnest, daughter of Dr. J. G. Earnest of this city. They left for an extended trip North, and the best wishes of The Journal goes with them.

THE Atlanta Society of Medicine is in a more flourishing condition than ever. It now occupies elegant new rooms in the English-American building, and meets regularly every first and third Thursdays in each month. The profession is always cordially invited.

---

THE Atlanta College of Physicians and Surgeons has opened in a most flattering manner. It already has over 200 students, and many more are expected during the first half the term. Dr. Westmoreland opened the College on October 5th, with by operating in the amphitheater. All the Clinics are held daily from 12 to 2 P. M., except that of Dr. Dunbar Roy, which is held on Tuesdays and Thursdays from 3 to 4:30 P. M.

---

DR. J. H. HALL, of Milledgeville, Ga., died of Bright's disease in that city on September 20th, 1898.

---

DR. B. N. HERRING, of Decatur, Ga., formerly of Clarkesville, Tenn., died in the former city, September 19th, at the age of twenty-six.

---

DIED.—R. L. Y. Long, of Newnan, Ga., at his home on October 7th. Dr. Long was one of the prominent physicians in the State.

---

DRS. POWELL & MARCHMAN, of Villa Rica, Ga., have formed a partnership in the practice of medicine and in the drug business. Both are well-known physicians, and the combination is a good one.

---

EACH year, the Georgia Legislature has among its membership a number of prominent physicians from different parts of the State. In looking over the list of members of the next legislature, we find that there is only one physician in the Senate, Dr. George C. Daniel, of Madison. In the House of Representatives we note the following: Dr. E. W. Watkins, of Gilmer county; Dr. C. H. Turner, of Rockdale county; Dr. George C. Erwin, of Towns county; Dr. G. W. Drawdy, of Wayne county.

---

Sir William Henry Broadbent has been appointed Physician Extraordinary to the Queen, in succession to the late Sir Richard Quain.

---

The *Medical Bulletin* informs us that Dr. Cowan, of Radford, Va., has been appointed Professor of Operative Surgery and Surgical Anatomy at the New York School of Clinical Medicine.

---

Dr. E. Emmet Reid, according to the *Philadelphia Medical Journal*, of the Johns Hopkins University, has been appointed Professor of Chemistry and Physics, in the Medical College of Charleston, S. C.

---

Dr. Claudius H. Martin, of Mobile, Ala., one of the South's distinguished surgeons, and a man who has occupied a prominent place in the various professional deliberative bodies, died in that city on October 3d.

---

Dr. Kathrina Van Tussenbrock has been appointed to the Professorship of the chair of Gynecology in the University of Utrecht. This shows that the women are coming to the front all over the world.

---

Dr. Jno. B. Hamilton, the gifted editor of the *Journal of the American Medical Association*, has been elected to the presidency of the Chicago Public Library. We have no doubt but that the library will flourish just as the *Journal* has under such able management.

MR. OTTO YOUNG, whose son recently died of tuberculosis, is building in Chicago, a hospital for tuberculous patients, at an estimated cost of $65,000. It will accommodate 75 patients, and will be provided with every device for the treatment of the disease by modern methods.

---

DR. PHINEAS CONNOR, of Cincinnati, is a member of the Board of Investigation recently appointed by President McKinley, for the purpose of investigating the alleged mismanagement of the medical department during the recent Spanish-American war. Dr. Keen was first selected, but declined to serve.

---

THE *Baltimore American* says that Porto Rico can offer no inducements to medical men. There is plenty of sickness there, but each native practices on himself. They are great believers in the efficiency of herbs, and these are used by the rich and poor alike. The editor thinks that for the graduates of the Eclectic schools this is indeed an El Dorado.

---

DR. JAMES TYSON has been elected for this session to fill the Chair of Practice of Medicine in the University of Pennsylvania. On account of the physical indisposition of Dr. John Ashurst, in the same institution, Dr. J. William White will have full charge of the Chair of Surgery. It seems to be the general impression that Dr. Tyson will fill this position permanently.

---

THE *American Medico-Surgical Bulletin* says that some unknown New York millionaire has made a gift of one million six hundred thousand dollars to Cornell University with which to start another medical college in New York city. Land has been bought on First avenue and Twenty-seventh street, and plans are ready for the erection of a six hundred thousand-dollar building.

---

THE daily papers inform us that Dr. Wetmore, Superintendent of Insane Asylum at Topeka, Kansas, has tendered his resignation to the Governor of that State. The reason given is that the management is incompetent and that a State "joint" exists at the asylum. The Doctor further charges all manner of inhumanity and debauchery by the members who compose the State Board of Charities.

---

THE American Microscopical Society, at its recent annual session, elected the following officers for the ensuing year: President, Dr. William C. Krauss, of Buffalo; First Vice-president, Professor A. M. Bleile, of Columbus, O.; Second Vice-President, Dr. G. C. Huber, of Ann Arbor, Mich.; Secretary, Professor Henry D. Ward, of Lincoln, Neb.; Treasurer, Magnus Pflaum, of Pittsburg; Executive Committee, Professor S. H. Gage, of Ithaca; Dr. A. Clifford Mercer, of Syracuse, and Dr. V. A. Moore, of Ithaca.

---

THE following officers were elected at Nashville of the Mississippi Valley Medical Association: President, Dr. Duncan Eve, Nashville, Tenn.; First Vice-President, Dr. A. J. Ochsner, Chicago, Ill.; Second Vice-President, Dr. J. C. Morfit, St. Louis, Mo.; Secretary, Dr. Henry E. Tuley, Louisville, Ky. (111 W. Ky. St.); Treasurer, Dr. Dudley S. Reynolds, Louisville, Ky. Next place of meeting, Chicago. Chairman of Committee of Arrangements, Dr. Harold N. Moyer. Time of meeting, October, 1899, date to be determined by the executive officers and the Chairman of the Committee of Arrangements.

---

THE following are the officers for the ensuing year of the American Association of Obstetricians and Gynecologists:

President, Edward J. Ill, M.D., Newark, N. J.; Vice-Presidents, Edwin Ricketts, M.D., Cincinnati, Ohio, and A. B. Miller, M.D., Syracuse, N. Y.; Secretary, William Warren Potter, M.D., Buffalo, N. Y.; Treasurer, X. O. Werder, M.D., Pittsburg, Pa.; Executive Council, A. Vander Veer, M.D., Albany, N. Y.; L. S. McMurtry, M.D., Louisville, Ky.; W. E. B. Davis, M.D., Birmingham, Ala.; John Milton Duff, M.D., Pittsburg, Pa.; L. H. Dunning, M.D., Indianapolis, Ind., and Walter B. Chase, M.D., New York.

WESTERN SURGICAL AND GYNECOLOGICAL ASSOCIATION.—The eighth annual meeting of the Western Surgical and Gynecological Association will be held at Omaha December 28 and 29, 1898. Titles of papers from some of the leading surgeons of the West are already in the hands of the secretary and the coming meeting promises to be the most interesting yet held. The local Committee of Arrangements at Omaha is actively preparing for the entertainment and comfort of those who attend. Surgeons and gynecologists, and those interested in the progress of these specialties, are cordially invited to affiliate themselves with us. The secretary will be glad to send application blanks. Titles of papers should be sent to the secretary as soon as possible, but not later than November 20, to insure a place on the program. Geo. H. Simmons, Secretary, Lincoln, Neb.; D. S. Fairchild, President, Clinton, Ia.

---

ARE THERE TOO MANY DOCTORS?—Under this title a correspondence, signed "Traveler," appeared in the *Post Dispatch* of September 22, 1898. The correspondence was in answer to the query of a young man in a previous issue of the paper regarding the advantages of a "Medical Night School." The correspondence is worthy of preservation. Here it is:

"One of your young men readers seems to desire the advantages (?) of a medical night school. If he investigates he will find that there are now two doctors for every one required, and that many medical schools are the bane of the medical profession and the laughing-stock of intelligent people. I have traveled through Kansas, Missouri (including St. Louis) and Illinois, calling on physicians, and have felt only regret for very many of them. The average income of a physician is not that of a good clerk and his expense is much heavier. Many doctors of ability and education leave the profession each year.

"As for medical schools, Missouri has now eighteen (more than the whole of Russia, with 105,000,000 people), and if the young man is as ambitious as his signature indicates, he will not find much difficulty in getting a sheepskin which afterwards will appear to him like the proverbial 'white elephant.' 'What will he do with it?'

"The advice of all conscientious physicians to young men, many of whom are utterly unfitted for it, who desire to study medicine should be, 'don't.' No, we don't need more medical schools, but we do need fewer, and those good ones."—*Medical Review.*

---

The Southern Surgical and Gynecological Association.—The eleventh annual meeting of the Association, which was announced to be held in Memphis, Tenn., Tuesday, Wednesday and Thursday, November 8th, 9th and 10th, has been postponed till Tuesday, Wednesday and Thursday, December 6th, 7th and 8th, 1898, on account of the quarantine regulations in some parts of the South. The Gayoso House has been selected as headquarters for the Association.

The following is a partial list of the papers to be read:

1. President's Address, Richard Douglas, M.D., Nashville, Tenn.

2. Gunshot Wounds, W. E. Parker, M.D., New Orleans, La.

3. Electro-therapeutics in Medicine and Surgery, James McF. Gaston, M.D., Atlanta, Ga.

4. The Normal Position of the Uterus Defined, A. H. Buckmaster, M.D., Charlottesville, Va.

5. Abdominal Opening for Intraperitonel Surgical Work, Jos. Price, M.D., Philadelphia, Pa.

6. The Choice of Material for Ligatures and Sutures in Gynecological Surgery, L. S. McMurtry, M.D., Louisville, Ky.

7. Repair in Cases of Complete Tear of the Perineum, Howard A. Kelly, M.D., Baltimore, Md.

8. Conservative Treatment of the Diseased Ovary, Jos. Taber Johnson, M.D., Washington, D. C.

9. Thoracotomy for Tumors Involving the Ribs, F. W. Parham, M.D., New Orleans, La.

10. The Use and Abuse of Normal Salt Solution, J. W. Bovee, M.D., Washington, D. C.

11. A Report of Fifty Prostatectomies, with Remarks on the Treatment of Prostatic Overgrowth in the Aged, John P. Bryson, M.D., St. Louis, Mo.

12. Remarks on the Surgery of the Gall-bladder and Bile-ducts, A. V. L. Brokaw, M.D., St. Louis, Mo.

13. Past and Present Surgery of the Gall-bladder and Bile-ducts, Wm. H. Myers, M.D., Fort Wayne, Ind.

14. The Pelvic Floor, Its Functions, Injuries and Repair, M. C. McGannon, M.D., Nashville, Tenn.

15. When Should we Operate for Appendicitis? A. M. Cartledge, M.D., Louisville, Ky.

16. Ureteral Anastomosis, Geo. H. Noble, M.D., Atlanta, Ga.

17. Ovarian Cysts as a Complication of Pregnancy and Labor, J. W. Long, M.D., Salisbury, N. C.

18. Incised Wounds of the Larynx, Edwin Walker, M.D., Evansville, Ind.

19. Tubal Pregnancy: Primary Rupture into the Broad Ligament, and Secondary into the Peritoneum; Laparotomy, Convalescence Complicated by Septic Diarrhea and Metastatic Abscess of the Liver, R. Matas, M.D., New Orleans, La.

20. Removal of Partially Descended, Infected, Strangulated Testicle, Complicated by Hernia, R. R. Kime, M.D., Atlanta, Ga.

21. The Diagnosis of Tubercular Peritonitis and Indications for Surgical Treatment, W. L. Robinson, M.D., Danville, Va.

22. Foreign Bodies in the Esophagus, with Report of Cases, A. Vander Veer, M.D., Albany, N. Y.

23. Penetrating Wounds of the Abdomen, Floyd W. McRae, M.D., Atlanta, Ga.

24. The Management of Pregnancy Complicating Intra-abdominal Tumors, with Cases, Rufus B. Hall, M.D., Cincinnati, O.

25. The Rarity of Ovarian Tumors in Negresses, I. S. Stone, M.D., Washington, D. C.

26. Tumors of the Breast, W. F. Westmoreland, M.D., Atlanta, Ga.

27. Penetrating Wounds of the Chest, J. B. Murfree, Murfreesboro, Tenn.

28. Surgery of the Pelvic Organs without Speculums or Retractors, W. H. Wathen, M.D., Louisville, Ky.

29. Report of a Case of Splenectomy for Wandering Hypertrophied Spleen, Wyatt Heflin, M.D., Birmingham, Ala.

30. Celiotomy in the Treatment of Retroverted Pregnant Uterus when Incarcerated, Henry D. Fry, M.D., Washington, D. C.

31. Odds and Ends in Pelvic Surgery, Walter B. Dorsett, M.D., St. Louis, Mo.

32. Treatment of Pelvic Inflammation, Jas. A. Goggans, Alexander City, Ala.

33. Mechanical Aids in Intestinal Surgery, J. D. S. Davis, M.D., Birmingham, Ala.

34. The History of Myomectomy, Chas. P. Noble, M.D., Philadelphia, Pa.

35. Observations upon Cranial Operations, with Report of Cases, Wm. Perrin Nicholson, M.D., Atlanta, Ga.

36. Plastic Surgery in Gynecology, W. D. Haggard, Jr., Nashville, Tenn.

37. Ventrofixation for Retrodisplacements of the Uterus, R. J. Trippe, M.D., Chattanooga, Tenn.

38. Removal of Five-gallon Ovarian Cyst from Girl Seventeen Years Old, R. R. Kime, M.D., Atlanta, Ga.

39. Transpleural Hepatotomy by Resection of the Rib and Free Incision; Recovery, R. Matas, M.D., New Orleans, La.

40. Subject to be announced, W. S. Elkin, M.D., Atlanta, Ga.

41. Surgery of the Stomach, W. E. B. Davis, M.D., Birmingham, Ala.

Members of the medical profession are cordially invited to attend. Dr. R. B. Maury, of Memphis, is chairman of the committee of arrangements. Richard Douglas, M.D., President; W. E. B. Davis, M.D., Secretary.

